# A Systematic Review and Meta-Analysis of the Effects of Vitamin D on Systemic Lupus Erythematosus

**DOI:** 10.3390/nu17172794

**Published:** 2025-08-28

**Authors:** Samira El Kababi, El Mokhtar El Ouali, Jihan Kartibou, Abderrahman Lamiri, Sanae Deblij, Rashmi Supriya, Ayoub Saiedi, Juan Del Coso, Ismail Laher, Hassane Zouhal

**Affiliations:** 1 Health, Care and Sustainable Development Laboratory, High Institute of Nursing Professions and Health Techniques, Casablanca 20503, Morocco; s.kababi@ispitscasa.ac.ma (S.E.K.); a.lamiri@ispitscasa.ac.ma (A.L.); s.deblij@ispitscasa.ac.ma (S.D.); 2 Sports Science Research Team, Institute of Sports Sciences, Hassan I University, Settat 26002, Morocco; elmokhtar.elouali@uit.ac.ma; 3 Laboratory of Biology and Health, Department of Biology, Ibn Tofail University, Kenitra 14000, Morocco; jihan.kartibou@uit.ac.ma; 4 Department of Sports and Health Sciences, Academy of Wellness and Human Development, Faculty of Arts and Social Sciences, Hong Kong Baptist University, Kowloon Tong, Hong Kong 999077, China; 5 Department of Physical Education and Sport Sciences, Faculty of Humanities and Social Sciences, University of Kurdistan, Sanandaj 66177-15175, Iran; saeidi_as68@yahoo.com; 6 Sports Science Research Centre, Rey Juan Carlos University, 28032 Madrid, Spain; juan.delcoso@urjc.es; 7 Department of Anesthesiology, Pharmacology and Therapeutics, The University of British Columbia, Vancouver, BC V6T 1Z4, Canada; ismail.laher@ubc.ca; 8 International Institute of Sport Sciences du (2I2S), 35850 Irodouer, France; 9 Laboratoire Optimisation de la Performance Sportive (LR09SEP01), Centre National de la Médecine et des Sciences des Sports, Tunis 1004, Tunisia

**Keywords:** 25-hydroxyvitamin D, autoimmunity, bone mineral density, fatigue, immunomodulation, inflammatory biomarker

## Abstract

**Background and Objective:** Systemic lupus erythematosus (SLE) is a chronic autoimmune disease characterized by widespread inflammation and multisystem involvement, leading to substantial morbidity. Given the immunomodulatory role of vitamin D and its association with disease activity in SLE, supplementation has emerged as a potential therapeutic strategy. However, findings across individual studies remain inconsistent, underscoring the need for a systematic review and meta-analysis to synthesize the current evidence on vitamin D supplementation for this disease. Thus, this study aimed to conduct a systematic review and meta-analysis on the effects of vitamin D supplementation on disease activity among patients with SLE. **Methods:** Systematic searches were carried out in four electronic databases (PubMed, Scopus, Web of Science, and Science Direct) with only studies published after 2013 as a restriction for the search strategy. An assessment of the included studies was conducted according to the recommendations of the Cochrane Handbook for Systematic Reviews of Interventions, using the risk of bias assessment tool in Review Manager (Revman) version 5.3. Included studies were randomized trials with vitamin D supplementation in patients with SLE and with pre–post intervention measures of disease activity. Meta-analyses were performed using random-effects models to estimate mean differences with 95% confidence intervals (CIs). Heterogeneity was evaluated using the *I*^2^ test, and sensitivity analysis and publication bias assessment were also performed. **Results:** A total of 186 articles were retrieved, of which 21 studies met the inclusion criteria. These studies had a combined sample size of 3177 adult participants and were conducted across 16 different countries. Regarding the impact of vitamin D supplementation on SLE patients, twelve (n = 12) studies reported positive associations, including reduced disease activity and improvements in clinical and laboratory parameters such as inflammatory markers, fatigue, and bone mineral density. In contrast, nine (n = 9) studies found no significant effects. In terms of meta-analytical data, our results indicate that, at the end of the supplementation, participants with vitamin D supplementation had significantly higher serum vitamin D levels compared to participants that receive a placebo (MD: 13.11 ng/mL; 95% CI: 8 to 19; *p* < 0.00001) despite comparable values before the onset of the supplementation. In addition, participants with vitamin D supplementation had lower scores in the Systemic Lupus Erythematosus Disease Activity Index (SLEDAI) compared to participants who received a placebo (MD: −1; 95% CI: −2 to −0.43; *p* = 0.002) despite comparable values before the onset of the supplementation. **Conclusions:** Our systematic review and meta-analysis suggest that vitamin D supplementation leads to a statistically significant reduction in SLEDAI scores, reflecting a meaningful decrease in disease activity. Given its immunomodulatory effects and favorable safety profile, vitamin D supplementation represents a simple and accessible adjunctive strategy that could support SLE management and improve patient outcomes in clinical practice.

## 1. Introduction

Systemic lupus erythematosus (SLE) is a chronic, inflammatory disease of connective tissue affecting the joints and many organs, including the skin, heart, lungs, kidneys, and nervous system [1]. The disease presents with different symptoms and predominantly affects women of childbearing age [2]. Dysfunction of the immune system leads to the production of autoantibodies directed against nuclear antigens, targeting various healthy tissues in SLE [3,4]. The deposition of immune complexes activates complement pathways and inflammatory cells, resulting in localized and systemic inflammation, leading to damage in multiple organs, particularly in the kidneys (lupus nephritis), skin (malar rash), and cardiovascular system, with significant morbidity and complications [4]. The causes of this immune system dysregulation are not fully understood, but predisposing factors include genetic, hormonal, and environmental factors such as medications (e.g., beta-blockers), ultraviolet radiation, certain viral infections, smoking, chemicals, and other industrial toxins [5].

The general pathophysiology of SLE is complex and not entirely clear. Tissue damage is facilitated by factors including the production of self-reactive dendritic cells, hyperactivity of the humoral immune system and cellular mediation, and the formation of deposits of nonspecific circulating immune complexes in some tissues [6]. Apoptotic defects, both quantitative and qualitative, are a major source of autoantigens [7]. However, the accumulation of apoptotic cells disrupts immune self-tolerance, triggering an increase in pro-inflammatory cytokines such as tumor necrosis factor-α (TNF-α) and interleukin-8 (IL-8) [8]. This process activates B and T lymphocytes, leading to the production of harmful autoantibodies and autoreactive T lymphocytes [1,9]. In addition, SLE is clinically unpredictable and is characterized by periods of remission and exacerbation, with a wide variety of clinical manifestations including constitutional signs and symptoms such as fatigue (80% to 100%), fever, and signs and symptoms related to organ involvement including the skin, liver, lungs, heart, and kidneys [10].

Diagnosis is based on clinical examination and laboratory tests. Serologic testing for anti-native DNA antibodies, anti-Smith antibodies (highly specific antibodies for some cases of SLE), anti-phospholipid antibodies, and inflammatory markers, as well as radiographic imaging, may be necessary to complete the diagnostic process [11]. Specifically, classification as SLE according to the Systemic Lupus International Collaborating Clinics (SLICC) criteria requires meeting at least four of seventeen criteria, including at least one clinical criterion and one immunologic criterion [12]. The therapeutic management of SLE includes general measures such as long-term treatment with hydroxychloroquine, the judicious use of corticosteroids during flare-ups, and, depending on the nature and severity of organ involvement, the possible use of immunosuppressive therapy and/or biologic agents [13].

Vitamin D deficiency has been linked to the expression, relapses [14], and pathogenesis of SLE [15] and was associated with higher disease activity [16]. Moreover, low serum vitamin D levels have been linked to several SLE comorbidities, including cardiovascular diseases [17], skin and kidney involvement [18], fatigue [19], and anti-dsDNA [20], as well as disease flares [21]. Vitamin D acts through a nuclear vitamin D receptor (VDR) that is present in most cells and regulates the transcription of over 200 genes with roles in neuromuscular and immune functions, the modulation of cell growth, and a reduction in inflammation [22]. The presence of VDRs and vitamin D activating enzyme 1, α-hydroxylase (CYP27B1) on cells involved in the immune response, such as dendritic cells, B lymphocytes, and T lymphocytes, provides evidence for its immunomodulatory properties [23]. The activation of VDRs promotes the differentiation of naive T cells into regulatory T cells (Tregs) and modulates the function of dendritic cells, promoting their ability to induce Treg development, inhibiting their potential to stimulate pro-inflammatory T cell responses (IL-6 and TNF-α) and increasing the production of anti-inflammatory cytokines (e.g., IL-10) [24]. Likewise, 1, 25-dihydrovitamin D3 inhibits dendritic cell maturation and expression of the IFN-α gene in patients with SLE [25]. A recent study reported that patients with vitamin D-deficient SLE had higher serum IFN-α activity and B-cell activation compared with those patients with higher vitamin D levels [26], while other studies demonstrated a higher prevalence of vitamin D deficiency in patients with SLE [20,24].

The results of studies on the benefits of vitamin D supplementation on several parameters in patients with SLE are contradictory. Several studies reported that vitamin D supplementation provides beneficial improvements in patients with SLE [17,25,26,27], while others failed to show that vitamin D supplementation improved various biomarkers in SLE patients [28,29,30,31,32,33]. The aim of our systematic review is to examine existing studies on the effects of vitamin D supplementation and SLE.

## 2. Materials and Methods

Our systematic review and meta-analysis were conducted in accordance with the guidelines outlined in the Cochrane Handbook for Systematic Reviews and Meta-Analysis of Interventions [34]. Furthermore, our literature search was executed following the standards set forth by the PRISMA (Preferred Reporting Items for Systematic Reviews and Meta-Analyses) statement [35].

### 2.1. Eligibility Criteria

Only studies investigating the impact of vitamin D supplements on the activity of systemic lupus erythematosus were included. We selected randomized and non-randomized studies that met the following criteria: (1) articles including patients diagnosed with SLE; (2) study design specified and country mentioned; (3) interventional studies with vitamin D supplementation; (4) studies with clear diagnostic criteria and well-defined vitamin D measurements and disease activity index (SLEDAI, ACR 1997 or SLICC 2012); (5) original studies published in scientific journals; (6) studies providing data to calculate a measure of an association (such as odds ratio, relative risk, or mean difference). We excluded studies that (1) were not published in English; (2) animal studies; (3) did not include validated methods for vitamin D assessment; (4) studies in children; (4) systematic or narrative reviews; and studies with a high risk of bias in terms of intervention or results.

### 2.2. Literature Search Strategy

Literature searches were carried out in four electronic databases (PubMed, Scopus, Web of Science, and Science Direct) with only studies published after 2013 as a restriction for the search strategy. The following key terms (and their synonyms) were included and used in combination with the operators “AND”, “OR”, “NOT”: CONCEPT 1 ((“Systemic lupus erythematosus” OR “SLE”) AND CONCEPT 2 (“Vitamin D” OR “25-hydroxyvitamin D” AND CONCEPT 3 (Autoimmune disease)).

### 2.3. Study Selection

Two investigators (SEK and EMEO) conducted a thorough review of the studies and a selection process based on predefined inclusion and exclusion criteria. Articles were initially selected based on their titles and abstracts. If an article met the eligibility criteria based on its abstract, the full text was then reviewed for further evaluation. In cases where the two investigators did not agree on the inclusion of an article, a consensus meeting involving all authors was convened to reach a resolution.

### 2.4. Data Extraction

Following the application of inclusion and exclusion criteria, data were independently extracted from each study in accordance with the PRISMA methodology and organized based on the PICOS framework (participants, interventions, comparisons, outcomes, and study design) (Table 1). Particular attention was given to details related to the intervention—vitamin D supplementation—including dosage, duration, and form of administration. Additional data extracted included the study name, objectives, results, country, population characteristics (e.g., average age, sex distribution, body mass index), disease duration, diagnostic criteria, baseline vitamin D levels, outcome measures, methods of assessment, and concomitant medications. All information was systematically compiled into summary tables to facilitate analysis.

### 2.5. Quality Assessment

We assessed the methodological quality of the selected studies using the Cochrane Handbook for Systematic Reviews of Interventions [34]. This assessment was based on seven criteria: adequate sequence generation, allocation concealment, blinding of participants and personnel, blinding of outcome assessment, incomplete outcome data, selective reporting of outcomes, and absence of other biases. Each criterion, as well as the overall risk of bias, was categorized into three levels: high risk of bias, unclear risk of bias, and low risk of bias. Based on these assessments, the overall quality of evidence was classified as high, moderate, or low risk of bias.

### 2.6. Data Analysis

The statistical analysis was conducted using the Cochrane Review Manager software (RevMan) 5.4.1. However, data on vitamin D levels and SLEDAI scores from the included studies, comparing the intervention and placebo groups, were analyzed using a random-effects model. The effect size was determined by calculating the mean difference (MD) with 95% confidence intervals (CIs). For each outcome, the MD for each study was calculated using mean and standard deviation values from groups supplemented with vitamin D and placebo before and after the supplementation and the sample size from each group. Additionally, heterogeneity across studies was evaluated using the *I*^2^ statistic. Following the classification of Higgins et al. [36], heterogeneity was categorized as follows: *I*^2^: 25–50% (moderate), 50–75% (substantial), and 75–100% (considerable). A statistical significance threshold of *p* < 0.050 was established, with values below this cutoff considered significant.

## 3. Results

### 3.1. Selection of Studies and Characteristics of Studies Included

**Figure 1:**
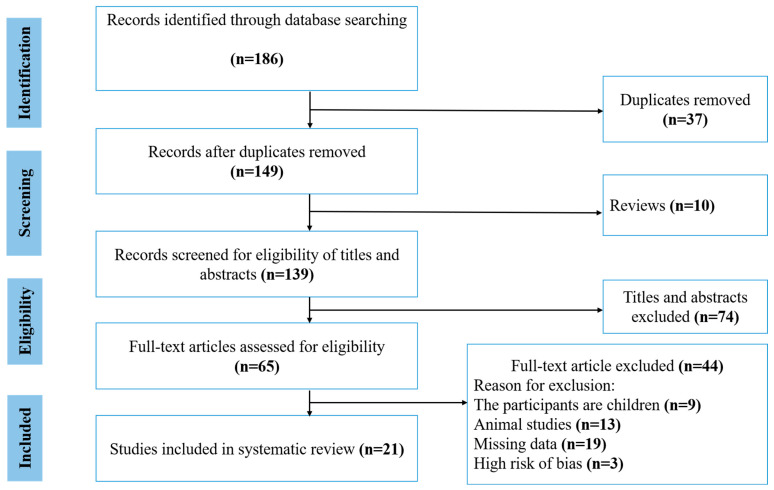
Flowchart of studies included in this systematic review.

Our literature search identified 186 publications. The initial screening process resulted in the removal of 37 duplicate studies, 10 review articles, and 74 articles deemed ineligible based on their title and abstract, leaving 65 articles to be examined in full text. After further review, we excluded 44 articles for the following reasons: 9 articles due to the population category, 13 were animal studies, 19 had missing data, and 3 had a high risk of bias. Finally, after applying our inclusion and exclusion criteria and performing a quality assessment, we included 21 studies in our systematic review (Figure 1). The 21 studies in our systematic review included 3177 participants, including men and women, were conducted across 16 different countries: Australia, the USA, Japan, Egypt, Paraguay, India, Thailand, Iran, Brazil, Malta, Spain, Tunisia, Saudi Arabia, Italy, Romania, and Indonesia. Of these, one study focused exclusively on men, nine studies focused exclusively on women, and twelve studies included both men and women. The general characteristics of the 21 studies included in our systematic review are summarized in Table 2.

### 3.2. Assessment of Study Quality

**Figure 2:**
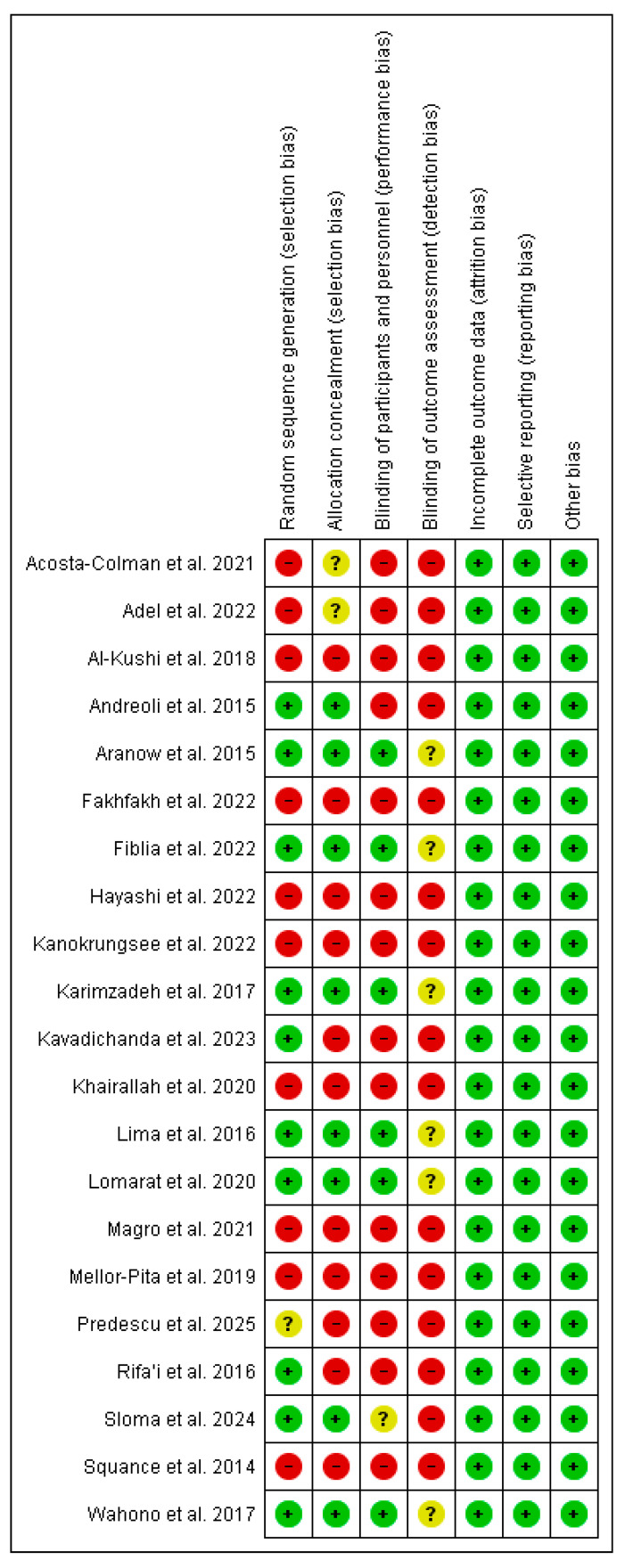
Risk of bias assessment of the included studies using the Cochrane RoB 2 tool [34]. Green: “low risk” (+), Yellow: “unclear risk” (?), and Red: “high risk” (−) [30,32,33,37,39,40,41,42,43,44,45,46,47,48,49,50,51,53].

**Figure 3:**
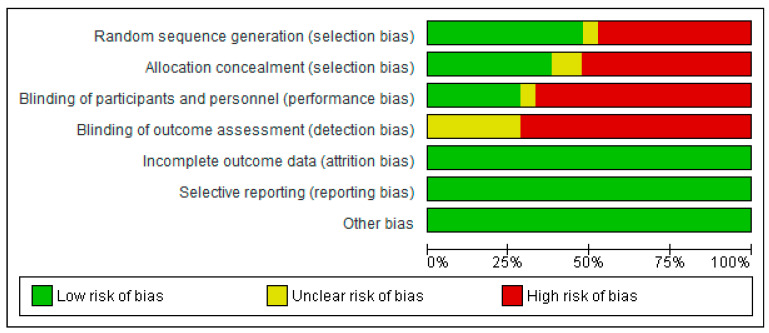
Summary of risk of bias judgments in included studies, displayed as percentages for each domain assessed using the Cochrane RoB 2 tool [34].

Of the studies included in the systematic review and meta-analysis, eight were rated as high quality, five as moderate quality, and eight as low quality. These lower quality ratings were mainly attributed to methodological limitations, including a lack of randomization and participant blinding. Despite these issues, the overall methodological quality of the included studies was considered satisfactory (Figure 2 and Figure 3).

### 3.3. Effect of Vitamin D Supplementation on Serum 25-OH Vitamin D

#### 3.3.1. The Results of the Systematic Review

The analysis of twenty-one studies indicates the following: (i) twelve (n = 12) studies reported significant differences between the vitamin D supplement and control groups, suggesting a beneficial effect of vitamin D supplementation on SLE disease [33,40,41,42,43,44,45,46,47,48,49,50,51]. (ii) Nine (n = 9) studies reported no significant differences between subjects receiving vitamin D supplementation and those who did not receive it [27,30,32,34,35,36,52,53]. Overall, the evidence suggests that vitamin supplementation has a positive effect on patients with SLE. However, these results demonstrate benefits on numerous clinical and biological parameters, including increased serum vitamin D levels, decreased inflammatory markers, decreased fatigue, improved bone mineral density, and decreased disease activity.

#### 3.3.2. The Results of the Meta-Analyses

**Figure 4:**
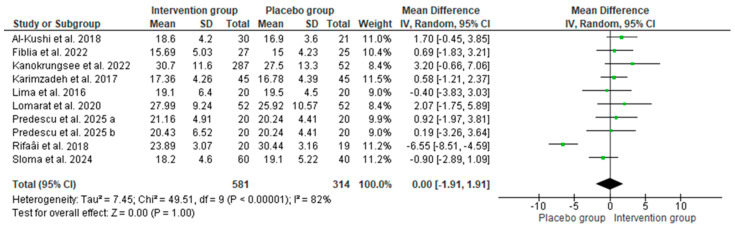
Baseline vitamin D levels between the intervention and placebo groups among patients with SLE. Forest plots show mean differences with 95% confidence intervals (CIs) between groups. The diamond at the bottom represents the pooled effect [27,33,41,43,44,47,50,51,52].

**Figure 5:**
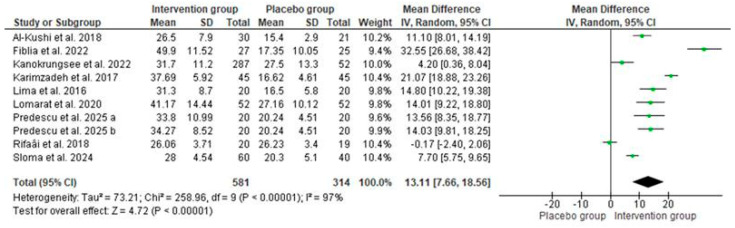
Vitamin D levels after supplementation with vitamin D in the intervention and placebo groups among patients with SLE. Forest plots show mean differences with 95% confidence intervals (CIs) between groups. The diamond at the bottom represents the pooled effect [27,33,41,43,44,47,50,51,52].

Ten studies [27,33,34,40,42,43,45,47,48,50] were used to analyze the effect of vitamin D supplements on 25(OH)D serum levels and on SLEDAI in SLE patients. Pooled data showed no statistically significant difference in baseline vitamin D levels between the intervention (i.e., those who received vitamin D supplementation) and placebo groups (MD: 0 ng/mL; 95% CI: −2 to 2; *p* = 1.00, with 82% of heterogeneity, Figure 4), confirming that participants across studies started with comparable vitamin D status. In contrast, after supplementation, the intervention group showed higher serum vitamin D levels compared to the placebo group (MD: 13.11 ng/mL; 95% CI: 8 to 19; *p* < 0.00001, with 97% of heterogeneity, Figure 5).

**Figure 6:**
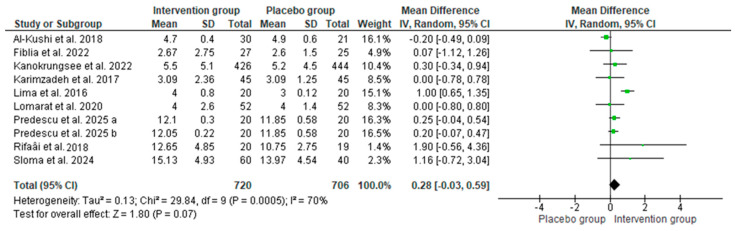
SLEDAI scores before vitamin D supplementation in intervention and placebo groups among patients with SLE. Forest plots show mean differences with 95% confidence intervals (CIs) between groups. The diamond at the bottom represents the pooled effect [27,33,41,43,44,47,50,51,52].

**Figure 7:**
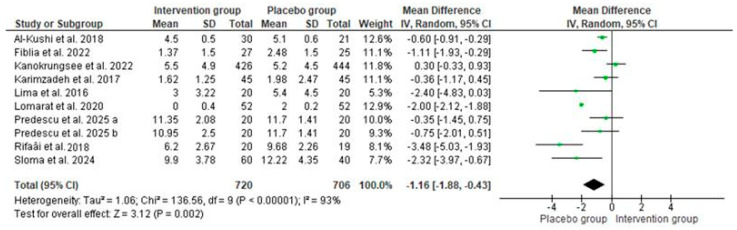
SLEDAI scores after vitamin D supplementation in intervention and placebo groups among patients with SLE. Forest plots show mean differences with 95% confidence intervals (CIs) between groups. The diamond at the bottom represents the pooled effect [27,33,41,43,44,47,50,51,52].

Regarding SLE disease activity, as measured by the SLEDAI score, the pooled data showed similar baseline disease activity in both groups (MD: 0; 95% CI: 0 to 1; *p* = 0.07, with 70% of heterogeneity, Figure 6). However, patients receiving vitamin D supplementation (intervention group) reported lower values of SLEDAI than those in the placebo group (MD: −1; 95% CI: −2 to −0.43; *p* = 0.002, with 93% of heterogeneity, Figure 7). Thus, significant improvements in SLEDAI have been reported for vitamin D baselines below 20 ng/mL [41,44,52].

### 3.4. Sensitivity Analyses

We conducted several sensitivity analyses so as to assess the robustness of our results. Fixed-effects and random-effects models were applied based on the degree of heterogeneity observed in the included studies. We conducted an additional sensitivity analysis, given that the choice of model did not alter the direction or significance of the pooled effect estimates and that both models produced comparable between-group differences. Specifically, we used a “leave-one-study-out” approach by systematically removing one study at a time to assess its influence on the overall mean differences (MDs), *p*-values, and heterogeneity. This analysis revealed no significant changes in effect size or heterogeneity levels, suggesting that no single study disproportionately influenced the results. Collectively, these results confirm the stability and reliability of the meta-analysis results.

### 3.5. Publication Bias

We assessed potential publication bias by visual inspection of funnel plots, according to the recommendations of the Cochrane Handbook for Systematic Reviews. The plots exhibited symmetrical distributions, suggesting the absence of substantial bias in the studies included in the meta-analysis. Moreover, funnel plots for all analyses are available in the Supplementary Material (Figures S1–S4).

## 4. Discussion

This systematic review and meta-analysis assessed the effectiveness of vitamin D supplementation on disease activity among SLE patients. The main finding of our systematic study was the significant correlation between vitamin D levels after vitamin D supplementation and improvements in SLE SLEDAI in the majority (~60%) of the 11 studies included in our meta-analysis. The comparison of both forest plot analyses reveals a correlation between increased vitamin D levels and a reduction in SLEDAI scores. The improved vitamin D status in the supplemented group is associated with clinical benefits, as reflected by decreases in disease activity. The high heterogeneity observed in both analyses underscores the variability in study methodologies, study populations, disease duration, and supplementation dosages. Despite this variability, the overall effect remains statistically significant and favors vitamin D supplementation. These findings support the hypothesis that vitamin D may play a beneficial immunomodulatory role in the management of SLE.

Vitamin D, an essential fat-soluble nutrient playing a role in calcium homeostasis, also has important functions in the regulation of the immune system [54], where it modulates the innate immune response and suppresses the adaptive immune response by interacting with its intracellular vitamin D receptor (VDR) expressed on various immune cells [55]. Consistent with our results, vitamin D supplementation was shown to increase vitamin D serum levels, reduce inflammatory, hemostatic markers [41,47,51,56], and improve fatigue [29]. A study by Acosta et al. reported that supplementation with vitamin D doses equal to or greater than 2000 IU/day increased serum vitamin D levels and decreased disease activity scores in patients with SLE [37]. Additionally, Fiblia et al. [41] investigated the effects of cholecalciferol supplementation at a dose of 5000 IU/day for 12 weeks on SLE disease activity. Their findings revealed an increase in average vitamin D 25(OH) levels and a more significant reduction in disease activity in the intervention group compared to the placebo group [41]. Likewise, these results were confirmed by Rifai et al. [51] who reported that supplementation of patients with SLE with vitamin D at 1200 IU/day for 3 months increased mean levels in the vitamin D supplementation group and improved disease activity and degree of fatigue. However, low doses of vitamin D supplements may be insufficient to decrease the disease activity for SLE patients as found in studies of Mellor-Pitta et al. [49].

In contrast with our findings, other studies have found no effect of vitamin D supplementation on various parameters of SLE disease [28,29,30,31,32,33]. No significant correlations were reported between vitamin D supplementation and SLE disease activity in North American women [39]. Similar results were reported by AL Kushi et al., who indicated no significant correlations between vitamin D status and immune markers or disease activity values after six months of vitamin D supplementation [33]. Another study indicated that genetically determined SLE can negatively affect vitamin D and 25 Hydroxyvitamin D levels, suggesting a bidirectional relationship in which SLE not only results from but may also contribute to vitamin D deficiency [57].

Furthermore, according to another report, vitamin D supplementation led to improvements in inflammatory markers and antibody production, but no changes in disease activity scores were observed in this study [58]. Additionally, there were no correlations between 25(OH)D levels and changes in IFNα gene expression [39]. Furthermore, there were no improvements in disease activity or SLE serology in patients with SLE who received high doses of vitamin D [38]. These findings indicate that intensive therapy is safe and effective for increasing levels of vitamin D to its recommended levels, but the dosage regimens showed no improvements in disease activity or the serology of SLE patients [38].

In terms of disease duration, we noticed that the majority of studies carried out on patients with long disease duration did not respond to vitamin D supplementation, and there was no improvement in disease activity [42,44,46], in contrast to early vitamin D supplementations in SLE patients who experienced decreases in disease activity, lower fatigue levels, and improved immunomodulatory effects in 40 juvenile-onset SLE patients with a disease duration ≤ 2.5 years [47]. These findings by Lima et al. (ref. [47] are consistent with our results. Studies by Rifai et al. [51] and Irfane et al. also reported that oral supplementation with vitamin D3 (cholecalciferol, 1200 IU/day or 30 mg/day) in women with SLE (aged 18–43 years, with disease duration ≤ 1 year, SLEDAI scores ≥ 5, hypovitaminosis D, and not using any vitamin D-containing medications) increased vitamin D levels in the intervention group and decreased SLEDAI scores. These findings suggest that early diagnosis and prompt treatment of SLE are critical to patient care and improved prognosis [59].

A longitudinal study of 409 relatives of patients with lupus, the majority of whom were asymptomatic and displayed 0 or 1 ACR criteria at baseline, reported that 11% transitioned to full-blown SLE after a mean follow-up of 6.4 years, compared to the 89% who did not develop SLE [60]. These findings suggest an imbalance between pro-inflammatory and anti-inflammatory cytokines and a greater variety of SLE-associated autoantibodies [61]. Additionally, these findings could also explain the conflicting results observed in some studies, where vitamin D supplementation was administered to patients diagnosed with SLE several years earlier (8–14 years) [33,39,42,44].

Recent evidence suggests that vitamin D supplementation in patients with SLE not only reduces SLEDAI scores but may also promote progression to remission or low-disease-activity states (LDASs), reducing the need for corticosteroids and modulating immunity [62,63]. Complete remission in SLE, as defined by the EULAR/ACR criteria, is characterized by an SLEDAI score of 0, a daily prednisone dose of ≤5 mg, the absence of clinically significant disease activity, no recent disease flares, and ongoing treatment with standard lupus therapies. Interestingly, just one study [27] reported a null score SLEDAI after vitamin D supplementation, indicating complete remission. Other studies [40,41,48] have reported a reduction in SLEDAI scores following vitamin D supplementation, with post-treatment values frequently falling below 4, a threshold commonly recognized for LDAS and considered a potential precursor to clinical remission. Although many of these studies lack complete data on corticosteroid dosage or the emergence of new symptoms, which are essential for confirming remission according to EULAR/ACR criteria, the observed improvements suggest that vitamin D supplementation may support the transition toward low disease activity or partial clinical remission in patients with SLE.

Based on the systematic review of the studies, high doses of vitamin D up to 50,000 IU/week (≈7000 IU/day) administered for 8 to 24 weeks appear to give the best clinical results on SLEDAI reduction [29,62]. Conversely, low doses (<1000 IU/day), as in the study performed by [38], do not show a significant effect on SLEDAI reduction. Moderate doses such as 2000 to 5000 IU/day also give beneficial results, as reported in the study by [40]. Accordingly, an effective daily dose between 5000 and 7000 IU/day (or 50,000 IU/week) for at least 8 to 12 weeks may be recommended to reduce disease activity in patients with systemic lupus erythematosus. However, these recommendations should be interpreted with caution, as no meta-analyses were performed by dosage due to the risk of underpowered or misleading interpretations due to heterogeneity in study designs and sample sizes.

Although vitamin D supplements are well tolerated, prolonged high doses can cause several adverse effects [64]. In the studies analyzed in this review, vitamin D supplementation at high doses (up to 50,000 IU/week, or ≈ 7000 IU/day) over periods ranging from 6 to 12 months was well tolerated by patients with SLE. None of these studies reported serious or clinically significant adverse events and several explicitly noted the absence of major adverse events associated with supplementation [38,50,52]. These findings are consistent with the general literature, which indicates that vitamin D is generally safe at daily doses of up to 4000 IU/day for most adults [64]. However, higher doses (5000–10,000 IU/day) may only be used in the short term under medical supervision to correct severe deficiency, but prolonged use beyond several months without laboratory monitoring may increase the risk of hypercalcemia, hypercalciuria, and renal impairment. At very high doses (>10,000 IU/day for several months), cases of toxicity have been documented [65,66]. However, another study carried out by McCullough et al. [67] reported that long-term supplementation with vitamin D3 in doses ranging from 5000 to 50,000 IUs/day appears to be safe for asthma, psoriasis, rheumatoid arthritis, rickets, and tuberculosis patients. The mean 25OHD levels on 10,000 IU/day were 96 ng/mL and 116 ng/mL, and no cases of hypercalcemia were observed using these doses of vitamin D for up to 7 years [67]. Taken together, considering both efficacy and safety data, a daily dose of 5000 to 7000 IU (or 50,000 IU/week) for a duration of 8 to 12 weeks appears to be a reasonable and potentially effective strategy for reducing disease activity in patients with SLE, provided that supplementation is monitored clinically.

In addition to the reduction in clinical activity measured by the SLEDAI score, vitamin D supplementation has an immunomodulatory effect. However, Wahono et al. [30], reported that the administration of 400 IU/day for 12 weeks resulted in a significant decrease in IL-6 and TGF-β1. Furthermore, it has been shown that changes in serum vitamin D levels can affect the proportion of Treg cell subset and TH17 cell subset and can also affect the levels of cytokines related to these T cell subpopulations [68]. These findings suggest that, beyond its impact on disease activity, vitamin D may also contribute to immune regulation in SLE, further supporting its therapeutic potential.

In addition to vitamin D status, SLE disease activity can also be influenced by environmental and biological factors [69]. It is possible that SLE patients with more active disease are prone to vitamin D deficiency as a result of renal insufficiency, the chronic use of medications that alter vitamin D metabolism, or reduced absorption that can be caused by glucocorticoids, anti-malarial and anti-convulsant medications, and the presence of anti-vitamin D antibodies [70]. Early initiation of therapy in the initial stages of SLE is crucial to addressing this significant unmet need [61]. This approach can help suppress inflammation, prevent irreversible tissue damage, and enhance the likelihood of a favorable response to treatment [61]. Previous studies reported that prescribing treatments to patients with SLE more than four years after diagnosis, particularly after the use of other immunosuppressants and glucocorticoids, may worsen long-term complications and complicate the interpretation of treatment responses [71]. However, additional studies and clinical trials are needed to better assess the effectiveness and safety of these therapeutic approaches so as to improve clinical outcomes and quality of life for patients with SLE.

Finally, our systematic review indicates that a predominance of studies (~60%) reported improvements in SLE disease activity after vitamin D supplementation. This finding may reflect studies with different study populations, small sample sizes, the dose of vitamin D supplementation, or methods for assessing the effects of vitamin D activity, and varying durations of SLE in different study populations. However, our meta-analysis reinforces the suggestion that vitamin D supplementation reduces SLE disease activity.

### Limitations

Despite the comprehensiveness of this systematic review and meta-analysis, we acknowledge several limitations. Although 22 studies were included in the review, only 11 met the inclusion criteria for meta-analysis, which may have limited the statistical power and precision of the pooled estimates. The remaining studies were synthesized qualitatively, which can introduce greater variability compared to a quantitative analysis. Furthermore, considerable heterogeneity existed in these studies regarding vitamin D dosages, treatment durations, and patient characteristics, hampering our ability to determine an optimal supplementation dose. Furthermore, other potentially influential factors, such as baseline vitamin D status, disease severity, concomitant use of other medications, and genetic or environmental differences, which were not consistently reported in these studies, may have also impacted treatment efficacy. These issues highlight the need for additional high-quality, large-scale randomized controlled trials with standardized vitamin D dosing regimens, longer follow-up periods, and consistent criteria for measuring SLEDAI. These trials should also take into account baseline vitamin D status, disease activity, and potential confounders such as concomitant medications, seasonal variations, and sun exposure. Furthermore, other studies are needed to explore the immunomodulatory pathways through which vitamin D may influence disease activity in SLE, particularly its effects on regulatory T cells, B cell function, and inflammatory cytokine profiles. Stratifying patients according to baseline 25(OH)D levels and monitoring immunological biomarkers alongside clinical outcomes may better identify subgroups likely to benefit most from supplementation.

## 5. Conclusions

Our research suggests that vitamin D supplementation may improve disease activity in patients with SLE. However, further studies are needed to determine optimal treatment schedules of vitamin D supplementation in the management of SLE.

## Figures and Tables

**Table 1:** PICOS (participants, interventions, comparisons, outcomes, study design).

PICOS Elements	Details
Participants	Patients diagnosed with SLE
Interventions	Monitoring vitamin D supplement dose in patients with SLE
Comparisons	A patient with vitamin D supplement compared to a patient without a vitamin D supplement (placebo)
Outcomes	Pre–post intervention changes in vitamin D levels and disease activity
Study designs	Case controls, cohorts, cross-sections, nRCTs, nRnCTs, RCTs

Abbreviations: nRCTs: randomized controlled trials, nRnCTs: non-randomized controlled trials.

**Table 2:** Characteristics of studies included in the systematic review.

Study	Country	Sample Size	Sex	Age (Means ± SD) or Range	Disease Duration (Years)	Supplement Duration	Vit D Supplement Doses	According to Diagnostic Criteria	Outcomes
Acosta-Colman et al. (2021) [37]	Paraguay	100	Men and women	27.5 ± 9.8	1.6	24 weeks	2000 IU/day	SLEDAI	Increasing levels of serum VD with supplementation (*p* = 0.0224), with a statistically significant association with disease activity
Adel et al. (2022) [32]	Egypt	38	Men and women	49.2 ± 8.1	6.2 ± 3.7	8 weeks	800 IU/day	SLEDAI	No significant differences in baseline vitamin D levels between the patients who were adherent or non-adherent to vitamin D supplementation (*p* = 0.1)
Al-Kushi et al. (2018) [33]	Saudi Arabia	81	Men and women	Gr 1: 36.4 ± 7.6 Gr 2: 35.2 ± 8.7 Gr 3: 37.7 ± 8.9	12.4 ± 3.4 13.9 ± 4.9 13.4 ± 2.9	6 months	1400 IU/day	SLEDAI	Vitamin D and calcium supplementation did not attenuate immune markers or disease activity but improved the bone mineral density in vitamin D-deficient SLE patients
Andreoli et al. (2015) [38]	Italy	34	Women	32.5 (19–44)	7 ± 2.3	24 months	25,000 IU/months	SLEDAI	Neither regimen of vitamin D supplementation affected SLE serology
Aranow et al. (2015) [39]	USA	54	Women	G1: 38.7 ± 12.27 G2: 36.5 ± 10.90 G3: 38.3 ± 12.88	10.1 ± 7.2	12 weeks	2000 IU/day Or 400 IU/day	ACR SLEDAI	No significant difference between patients receiving a vitamin D3 supplement and those receiving a placebo
Fakhfakh et al. (2021) [40]	Tunisia	106	Men and women	37.8 (21–63)		24 months	2000 IU/day	ACR SLEDAI	A significant difference in the mean levels of 25[OH]D between vitamin D-supplemented SLE patients and controls was observed but was not associated with changes in SLEDAI
Fiblia et al. (2022) [41]	Indonesia	60	Women	18–60 years	1–5 years	12 weeks	5000 IU/day	MEX- SLEDAI	Supplementation with cholecalciferol increased vitamin D levels and improved disease activity
Hayashi et al. (2022) [42]	Japan	870	Men and women	45 ± 14	12.75 ± 10.08		No data	ACR SLEDAI	Vitamin D supplementation did not change disease activity
Kanokrungsee et al. (2022) [43]	Thailand	414	Men	than 18 years	7 ± 3.4	3 months	10,000 IU of vitamin D2/week	SLEDAI-2K	Vitamin D replacement therapy increased serum vitamin D levels in 45% of patients
Karimzadeh et al. (2017) [44]	Iran	90	Men and women	IV: 33.78 ± 6.2 PB: 35.69 ± 6.8	IV: 9.53 ± 3.8 PB: 10.98 ± 3.5	12 weeks	50,000 IU/month	SLEDAI	The mean values of SLEDAI were not different before and after vitamin D supplementation in intervention and placebo groups
Kavadichand a et al. (2023) [45]	India	702	Men and women	29.44 ± 10.7	G 1: 1.6 G 2: 1.3	6 months	30,000 IU/Day	ACR SLEDAI	High-dose oral vitamin D supplementation may be safe and effective in improving vitamin D levels in SLE but had a weak correlation with disease activity
Khairallah et al. (2020) [46]	Egypt	100	Men and women	IV: 28.30 ± 8.9 PB: 25.32 ± 6.98	10 ± 3.2 years			SLEDAI	Vitamin D supplements do not appear to significantly decrease the positivity of anti-dsDNA and SLE activity
Lima et al. (2016) [47]	Brazil	40	Men and women	IV: 18.5 6 3.5 PB: 19.3 6 3.3	2.5 ± 1.5	24 weeks	50,000 IU/week	SLEDAI	Cholecalciferol supplementation decreased disease activity and improved fatigue in juvenile-onset SLE patients
Pakchotanon et al. (2020) [27]	Bangkok Thailand	91	Men and women	42.41 ± 13.25	6 ± 1.5	6 months	D2: 100,000 IU/ week/ 4 weeks 40,000 IU/Week For 20 weeks D3: 800 IU/day/24 week	ACR SLEDAI	Study was inconclusive in demonstrating the efficacy of high-dose ergocalciferol in controlling SLE disease activity
Magro et al. (2021) [48]	Malta	31	Women	47.9 ± 13.7	14.1 ± 8	12 months	vitamin D insufficiency: 8000 IU/day for 4 weeks/ followed by 2000 IU/day vitamin D deficiency: 8000 IU/day/8 weeks followed by 2000 IU daily	ACR SLIC SLEDAI	Improved disease activity and fatigue were noted
Mellor-Pita et al. (2019) [49]	Spain	47	Women	48.8 (21–65)	10.85 ± 7.9	3 months	400 to 800 IU/ day with 500 to 1000 mg of calcium	ACR SLEDAI	No significant association between 25(OH)D serum levels and cardiovascular risk factors and disease activity
Predescu et al. (2025) [50]	Romania	60	Men and women	3.23 ± 12.65	IV1: 9.3 ± 3.48 IV2: 9.83 ± 3.97 PB: 9.83 ± 3.97	6 months	IV1: 4000 IU/Day IV2: 8000 IU/Day	SELENA-SLEDAI	Significant increases in vitamin D levels and serum complement levels in the supplementation groups. Slight reduction in SELENA-SLEDAI scores in the treated groups
Rifa’i et al. (2018) [51]	Indonesia Malang	39	Women	IV: 28.25 ± 6.97 PB: 27.75 ± 6.86	˂1 years	3 months	1200 IU/day	ACR SLEDAI	Supplementation with vitamin D improved disease activity and degree of fatigue
Sloma et al. (2024) [52]	Romania	100	Women	IV: 26.80 ± 4.57 PB: 28.15 ± 5.99	IV: 3.86 ±1.78 PB: 4.25 ± 1.93	3 months	2000 IU/day	SLEDAI	The average SLEDAI score was reduced after three months of supplementation
Squance et al. (2014) [53]	Australia	80	Women	PB: 49.8 ± 12.4 IV: 47.7 ± 13.5	7.7 ± 6.2 years	3 months		ACR	Vitamin D supplementation along with regular monitoring should be a consideration as part of individual patient health management plans
Wahono et al. (2017) [30]	Indonesia	40	Women	IV: 29.1 ± 8.95 PB: 30.3 ± 10.0	2.12 ± 1.5	3 months	400 IU/day	ACR SLIC SLEDA	No significant differences in SLEDAI reduction, decreased serum levels of IL-6, and increased levels of TGF-β1 serum among groups

SLE: systematic lupus erythematous, ACR: American College of Rheumatology, SLEDAI: Systemic Lupus Erythematosus Disease Activity Index, PB: placebo group, and IV: interventional group.

## Data Availability

All the data used can be found in the manuscript.

## Supplementary Materials

The following supporting information can be downloaded at: https://www.mdpi.com/article/10.3390/nu17172794/s1, Figure S1: Funnel plot of studies comparing baseline vitamin D levels in intervention and placebo groups. Figure S2: Funnel plot of studies comparing vitamin D levels after supplementation in intervention and placebo groups in patients with SLE. Figure S3: Funnel plot of SLEDAI scores before vitamin D supplementation in intervention and placebo groups. Figure S4: Funnel plot of SLEDAI scores before vitamin D supplementation in intervention and placebo groups.
