# Supplementary material for: A Systematic Review and Meta-Analysis of the Effects of Vitamin D on Systemic Lupus Erythematosus
Source: Nutrients. 2025 Aug 28;17(17):2794. doi: 10.3390/nu17172794 (PMC12430488; doi:10.3390/nu17172794)
Supplement: Supplementary file 1 [file nutrients-17-02794-s001.zip › nutrients-3706373-supplementary.pdf]

Supplementary Material

Funnel plots

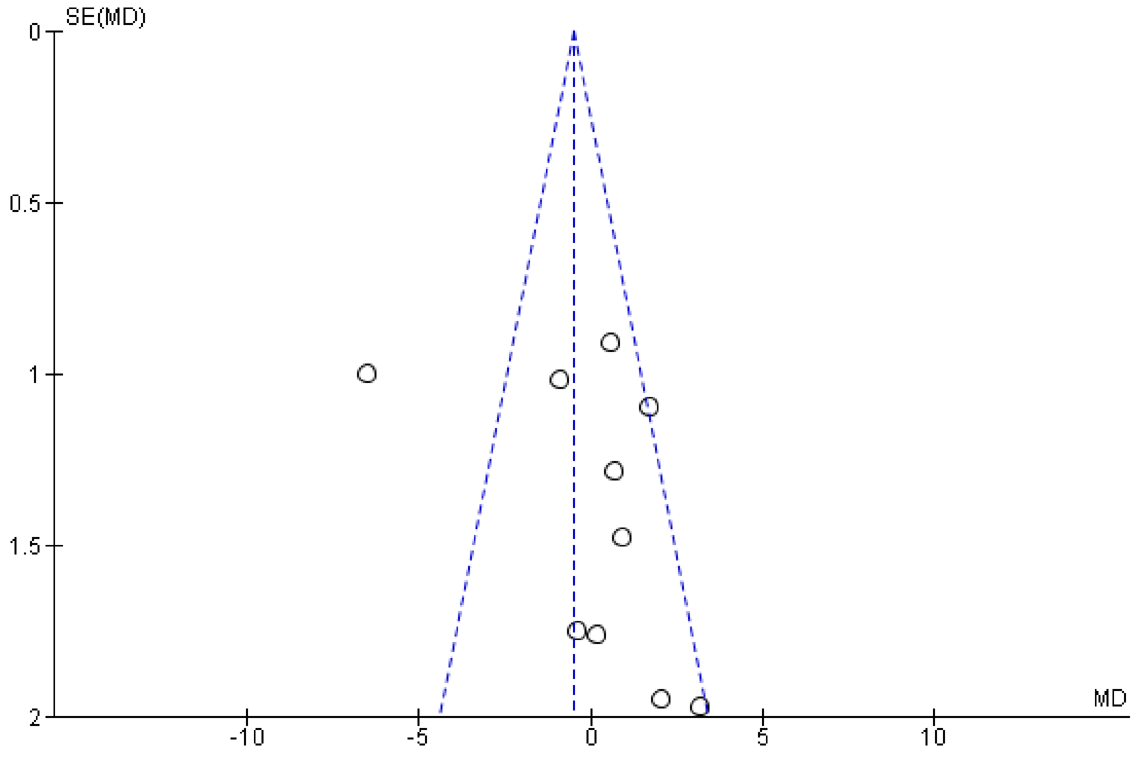

Figure S1: Funnel plot of studies comparing baseline vitamin D levels in intervention and placebo groups.

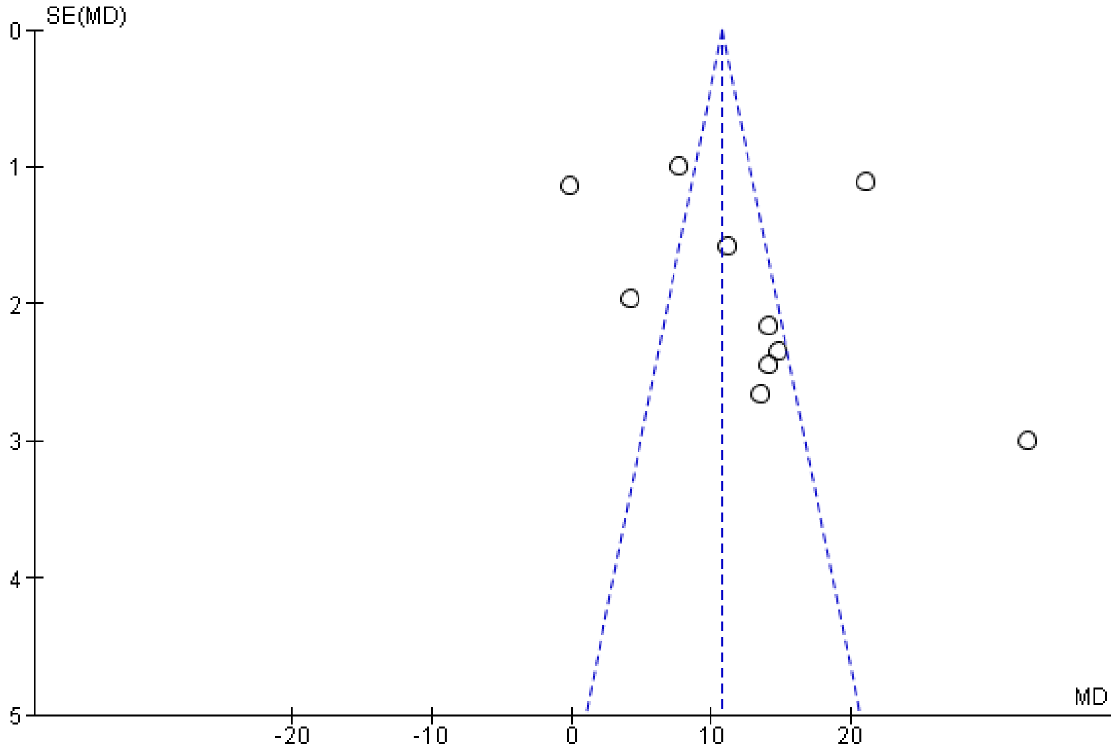

Figure S2: Funnel plot of studies comparing vitamin D levels after supplementation in intervention and placebo groups in patients with SLE.

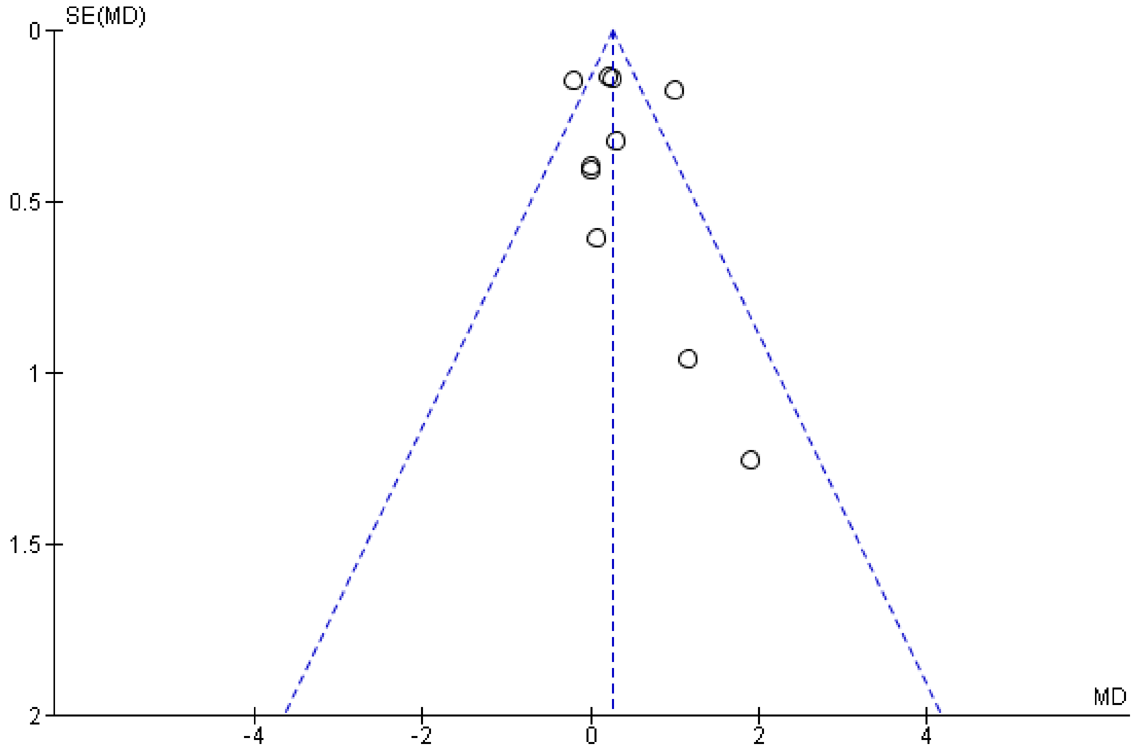

Figure S3: Funnel plot of SLEDAI scores before vitamin D supplementation in intervention and placebo groups.

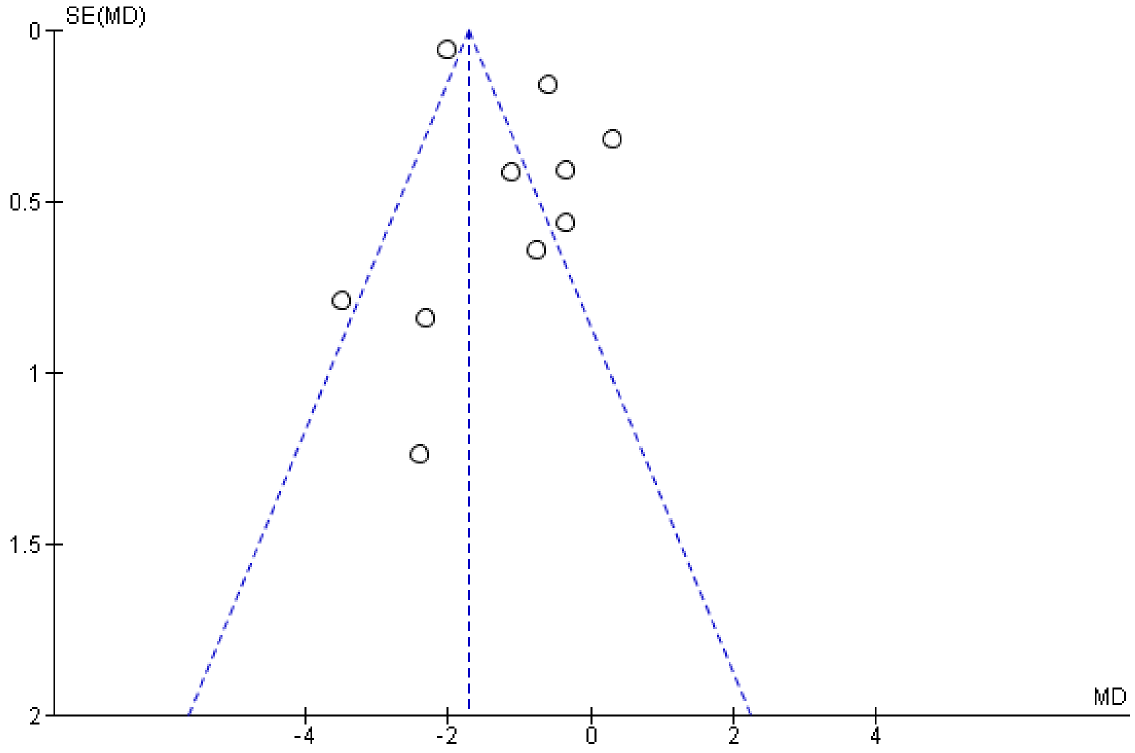

Figure S4: Funnel plot of SLEDAI scores after vitamin D supplementation in intervention and placebo groups.
